# Supplementary material for: Circulating sLR11 levels predict severity of pulmonary hypertension due to left heart disease
Source: PLoS One. 2021 Dec 29;16(12):e0261753. doi: 10.1371/journal.pone.0261753 (PMC8716052; doi:10.1371/journal.pone.0261753)
Supplement: S1 Table — (DOCX) [file pone.0261753.s001.docx]

**S1 table**

**Single variable.**

|  | **HR** | **95% CI** | **p-value** |
| --- | --- | --- | --- |
| **BNP** | 1 | 1.00 to 1.00 | 0.25 |
|  |  |  |  |
| **sLR11** | 1.07 | 0.90 to 1.26 | 0.46 |
|  |  |  |  |
| **Creatinine** | 1.2 | 0.86 to 1.67 | 0.27 |
|  |  |  |  |
| **age** | 1.09 | 0.97 to 1.21 | 0.14 |
|  |  |  |  |
| **sex** | 0.49 | 0.05 to 4.39 | 0.52 |
|  |  |  |  |
| **BMI** | 1.02 | 0.79 to 1.32 | 0.86 |
|  |  |  |  |
| **HT** | 359500000 | 0 to Infinity | 1 |
|  |  |  |  |
| **DL** | 0.95 | 0.16 to 5.69 | 0.96 |
|  |  |  |  |
| **DM** | 2.58 | 0.43 to 15.47 | 0.3 |

**Combination variables.**

|  | **HR** | **95% CI** | **p-value** |
| --- | --- | --- | --- |
| **BNP** | 1.00 | 1.00 to 1.00 | 0.25 |
| **sLR11** | 1.05 | 0.83 to 1.32 | 0.71 |
| **Creatinine** | 1.77 | 0.42 to 7.41 | 0.43 |
| **age** | 1.25 | 0.88 to 1.78 | 0.21 |
| **sex** | 0.06.48 | 0.00 to 70.8 | 0.44 |
| **BMI** | 1.05 | 0.67 to 1.64 | 0.83 |
| **HT** | 328000000 | 0.00 to Infinity | 1.0 |
| **DL** | 1.98 | 0.06 to 71.75 | 0.71 |
| **DM** | 0.885 | 0.07 to11.05 | 0.92 |

Cox proportional hazard analysis was performed for HF event by explanatory variables that sLR11, BNP, age, sex, BMI, histories of hypertension, dyslipidemia, diabetes and creatinine. There were not significantly different each single variable and combination variables.
