# Supplementary material for: Circulating sLR11 levels predict severity of pulmonary hypertension due to left heart disease
Source: PLoS One. 2021 Dec 29;16(12):e0261753. doi: 10.1371/journal.pone.0261753 (PMC8716052; doi:10.1371/journal.pone.0261753)
Supplement: Attachment — Submitted filename: PLOS ONE revise authors reply konishi (1).docx [file pone.0261753.s002.docx]

June 18, 2021

Manuscript ID　PONE-D-21-06716R1

Revised manuscript title:Circulating sLR11 levels predict severity of pulmonary hypertension due to left heart disease

Dear Editors and Reviewers

PLOS ONE

Thank you very much for reviewing our manuscript and offering valuable advice. We have addressed your comments with point-by-point responses, and revised the manuscript accordingly.　We performed additional important experiments, and modified the Abstract, Materials and Methods, Results and Discussion sections based on the comments provided by the reviewers. We have uploaded a copy of the original manuscript showing all revisions and the red characters are places of correction.

Sincerely,

Hakuoh Konishi, MD, PhD　Associate professor

Department of Cardiovascular Biology and Medicine, Juntendo University Graduate School of Medicine.

2-1-1 Hongo, Bunkyo-ku, Tokyo 113-8421, Japan

E-mail: yjyouki@juntendo.ac.jp

Tel: +81-3-3813-3111　Fax: +81-3-5689-0627

Responses to the Comments by the associate editor:

**Reviewer #1**: 
*Specific comments
1. sLR11 has been believed to be released from cell membrane, and not secreted from cells. All the described “secreted” should be corrected to “released” in the sections of introduction and discussion.*

Thank you for your recommendation, I changed the description from secreted to released.

*2. The* *description of BNP levels in “BNP was also higher in the high-sLR11 group (83.2±100.2 pg/ml) than in the normal-sLR11 group (576.6±1023.9 pg/ml).” should be replaced each other?*

Thank you for pointing out mistake. We collected this description.

**Reviewer #2:** 
*1. The main problem with this paper is that each statistical method involves a separate set of variables and the link between the results is not very clear. Perhaps the choice of selecting only some variables in each analysis is obvious by a medical viewpoint, but I -- as a statistician -- I would have welcomed an analysis were the selection of the variables under analysis was appropriately motivated. In particular, I was not able to understand why some variables were included in the multivariate regression analysis (e.g., age, BMI, Creatinine ...) and not in the subsequent survival analysis; or why the correlation analysis between sLR11 and pulmonary hemodynamics parameters was not adjusted for the baseline variables of Table 1. All these choices should be clarified in the text, as a courtesy to the reader who is not necessarily an expert of the field.*

Thank you for your recommendation. According to your excellent suggestion, we defined to the objective variable was mPAP and selected an explanation variable which is high in degree of influence on an objective variable that cardiovascular risk factor (age, sex, BMI, creatinine, histories of hypertension, dyslipidemia, DM and sLR11 or logBNP) and reanalyzed multivariate analysis. We assessed to each variable by bivariate analysis and confirmed that there was not a variable that had 0.95 multiple correlation coefficient > 0.95. mPAP was associated with higher levels of sLR11 and logBNP. Levels of sLR11 offered a significant predictor of mPAP, similar to log BNP (Table2).

**Table 2. Multivariate analysis of the factors associated with mPAP.**

|  | **B** | **SE B** | **β** | **p-value** |
| --- | --- | --- | --- | --- |
| **age** | **-0.03** | **0.1** | **-0.06** | **0.74** |
| **sex** | **5.43** | **3.43** | **0.34** | **0.13** |
| **BMI** | **0.18** | **0.43** | **0.08** | **0.67** |
| **Creatinine** | **-0.29** | **0.85** | **-0.06** | **0.74** |
| **Diabetes mellitus** | **2.3** | **3.75** | **0.13** | **0.55** |
| **Hypertension** | **3.73** | **2.96** | **0.23** | **0.22** |
| **Dyslipidemia** | **-1.17** | **2.95** | **-0.08** | **0.69** |
| **sLR11** | **0.75** | **0.31** | **0.45** | **0.02** |
|  | **F-statistic: 2.16** | | | |
|  | **Multiple R-squared: 0.22** | | | |
|  |  |  |  |  |
|  |  |  |  |  |
|  |  |  |  |  |
|  | **B** | **SE B** | **β** | **p-value** |
| **age** | **-0.17** | **0.1** | **-0.3** | **0.1** |
| **sex** | **5.56** | **3.22** | **0.34** | **0.1** |
| **BMI** | **0.36** | **0.39** | **0.16** | **0.36** |
| **Creatinine** | **-1.88** | **0.98** | **-0.39** | **0.07** |
| **Diabetes mellitus** | **7.12** | **3.29** | **0.4** | **0.04** |
| **Hypertension** | **4.01** | **2.8** | **0.25** | **0.16** |
| **Dyslipidemia** | **-0.94** | **2.79** | **-0.06** | **0.73** |
| **logBNP** | **7.54** | **2.4** | **0.61** | **0.004** |
|  | **F-statistic: 2.81** | | | |
|  | **Multiple R-squared: 0.47** | | | |

*2. Study design. The authors excluded 28 patients (about 40% of the available sample) who were followed-up for less than 5 years. Exclusion of right-censored subjects could severely bias the analysis. It is also a unnecessary waste of information, because the subsequent statistical analysis could be performed without excluding these subjects.*

We apologize for our unclear description of why excluded 28 patients. Excluded 28 patients were difficult to follow up or very short time because they were transferred or outpatients were transferred to another hospital after surgery and could not be tracked in the electronic medical records.

*3. Survival analysis. It looks like (not clear from text, please clarify) survival analysis has been performed by merging hospitalization for heart failure and all-cause death. This choice unnecessarily complicates the interpretation of the results. These two events should be kept separated. In addition, survival analysis relies on a log-rank test that compares survival of two groups, obtained after dichotomizing variable sLR11. Dichotomization of a continuous variable leads to an unnecessary waste of information and it is in general not recommended. The influence of sLR11 on survival can be studied by a Cox regression model that includes sLR11 as a continuous predictor.*

Thank you very much for your helpful recommendations. We performed survival analysis that was divided two events, hospitalization for heart failure and all cause of death. Hospitalization for heart failure was significantly higher in the event risk in the high sLR11 group after 5 years.　Also, all-cause mortality was observed in the high sLR11 group (2deaths), but no significant difference was observed. We have added figure3A, B, respectively. We analyzed Cox proportional hazard analysis was performed for each event selecting sLR11, BNP, age, sex, BMI, diabetes, dyslipidemia, history of hypertension, and creatinine as explanatory variables, however, there was not significantly different each single variable and combination. It might be caused by small scale and a few events.

*4. Multivariate linear regression. Table 2: I don't understand the meaning of the estimates B and beta. Are they the regression coefficients of univariate and multivariate regression models? Please clarify. Also please clarify whether the p-value is associated to beta or B. More importantly, there are two issues with this analysis. First, the models include many not significant covariates. This could be a clue of multicollinearity. Variables should be included after an appropriate analysis of multicollinearity. Second: only some variables have been included. What about the other baseline variables shown in Table 1? For example, age is included but gender is not included: why? The authors should at least run a variable selection algorithm to motivate the exclusion of some variables.*

Thank you for your help, we described what is the B and β that mean partial regression coefficient (B) and standard partial regression coefficient (β). We described above about multicollinearity.
