# Supplementary material for: Circulating sLR11 levels predict severity of pulmonary hypertension due to left heart disease
Source: PLoS One. 2021 Dec 29;16(12):e0261753. doi: 10.1371/journal.pone.0261753 (PMC8716052; doi:10.1371/journal.pone.0261753)
Supplement: Attachment — Submitted filename: response.docx [file pone.0261753.s003.docx]

Responses to Comments

Reviewer #1:

*We wish to express our deep appreciation to Reviewer #1.*

Reviewer #2:

*Thank you very much for providing important comments.*

1. Statistical analysis: “baseline characteristics….using two-sided　two-sample T-tests for normally distributed continuous variables”. Are　all baseline characteristics normally distributed? If not, how about　non-normally distributed variables?

*Thank you for raising this important issue. We have mentioned in the revised text that the Mann-Whitney U test was applied for the analysis of non-normally distributed continuous variables.*

2. Statistical Analysis: “Raw counts and percentages are described as mean and standard deviation”. Please clarify what this statement!

*What we meant was that data in the form of raw counts and percentages described in the text are mean values ± standard deviation (SD).*

3. Results: 28 out of 66 MR patients were excluded from the original enrolled sample. Are there any characteristics difference between these 28 and the remaining patients?

*The 28 patients were excluded on the basis that they could not be tracked in the electronic medical records. As a result, we do not have data with which to compare excluded and analyzed patients.*

4. Results: “Median age was 64.8±13.1”. So 64.8 is median age but what 13.1 is? IQR? Please clarify this. Similar question applies to other reported information.

*We apologize for this error. We have deleted the median value and provided the data as mean ± standard deviation.*

5. Results: There are many typos or errors here and there!! This is quite a surprising to see for a revised manuscript. Also, it might be benefit to use English editing as well. To name some examples, a. “there was not a variable that had 0.95 multiple correlation coefficient > 0.95.” Please clarify what this statement means?

*Our manuscript had undergone English proofreading before the first submission, but the revised text has undergone further proofreading.*

b. “We defined to the objective variable was mPAP and selected an explanation variable which is high in degree of influence on an objective variable that cardiovascular risk factor.” Please clarify what this statement means.

*Thank you for this comment. We have added a reference for the cardiovascular risk factor after this sentence.*

6. Results. “Based on our previous normal human sLR11 level of…, we established an upper limit of normal for sLR11 of 9.4..” Please provide more detail how this was establish? If this criteria is from previous work, please cite appropriate reference.

*We have cited our previous report (Ref. 15) showing that the mean (±SD) serum sLR11 level in healthy individuals was 7.8 ± 1.6 ng/mL.*

7. Tables 1 and 3. “data are presented as n,…mean,…or median.” Please make clear notes which variables were presented in mean and which in median.

*As noted in response to Comment 4, values have been provided as means ± standard deviations.*

8. Table 2. Please add degree of freedom information for the F-statistic. Otherwise, it doesn’t have any meaning with only F-stat.

*Thank you for this comment. We have added degrees of freedom to Table 2.*
